# Structure of the Cell-Wall-Associated Polysaccharides from the Deep-Sea Marine Bacterium *Devosia submarina* KMM 9415^T^

**DOI:** 10.3390/md19120665

**Published:** 2021-11-26

**Authors:** Maxim S. Kokoulin, Lyudmila A. Romanenko, Aleksandra S. Kuzmich, Oleg Chernikov

**Affiliations:** 1 G.B. Elyakov Pacific Institute of Bioorganic Chemistry, Far Eastern Branch, Russian Academy of Sciences, 159/2, Prospect 100 Let Vladivostoku, 690022 Vladivostok, Russia; lro@piboc.dvo.ru (L.A.R.); assavina@mail.ru (A.S.K.); chernikov@piboc.dvo.ru (O.C.); 2 School of Natural Sciences, Far Eastern Federal University, 8, Sukhanova Str., 690950 Vladivostok, Russia

**Keywords:** marine bacteria, deep sea, *Devosia*, polysaccharide, xylulose

## Abstract

Two cell-wall-associated polysaccharides were isolated and purified from the deep-sea marine bacterium *Devosia submarina* KMM 9415^T^, purified by ultracentrifugation and enzymatic treatment, separated by chromatographic techniques, and studied by sugar analyses and NMR spectroscopy. The first polysaccharide with a molecular weight of about 20.7 kDa was found to contain d-arabinose, and the following structure of its disaccharide repeating unit was established: →2)-α-d-Ara*f*-(1→5)-α-d-Ara*f*-(1→. The second polysaccharide was shown to consist of d-galactose and a rare component of bacterial glycans-d-xylulose: →3)-α-d-Gal*p*-(1→3)-β-d-Xlu*f*-(1→.

## 1. Introduction

Oceans cover about 70% of our planet, and deep-sea areas make up about 75% of the total ocean volume. Most of the deep environment is considered exceedingly harsh, with temperatures below 5 °C, extreme pressure, and no sunlight. Deep-sea organisms have had to evolve, often through unusual adaptations, to live, reproduce, and thrive in these unique conditions. Indeed, the depths of the sea seem to have considerable potential for the investigation of extremophilic microorganisms. In recent years, a growing number of various novel bacterial genera and species have been isolated and described [1]. They include strains able to produce a wide range of new molecules, e.g., diverse biologically active polymers [2] and low molecular weight metabolites [3]. A comprehensive study of these substances could lead to the discovery and characterization of innovative compounds and materials with properties desirable for pharmaceutical or industrial applications.

Among these substances, carbohydrate-containing biopolymers, a significant structural component of the bacterial envelope, are of increasing scientific interest. Bacterial polysaccharides can be secreted into the extracellular environment (exopolysaccharides, EPS) or be bound to the cell surface (so-called capsular polysaccharides, CPS) and represent a great reservoir of new molecules whose structures and functional properties are largely unknown. From a chemical point of view, these biopolymers are high molecular weight glycans in which structural variation is almost unlimited, and unusual sugars are often their components [2]. Bacterial glycans have a more than 10-fold greater diversity in the nature of monosaccharides, compared with eukaryotic polysaccharides. [4]. The structural versatility of bacterial polysaccharides is associated with a wide spectrum of biological functions and physicochemical properties, from structural reinforcement to adhesion, colonization, camouflage, and the concentration on the surface of definite molecules. In recent years, various marine bacterial EPS with biomedical properties (antioxidant, antitumor, immunomodulatory, wound healing, etc.) have been considered promising candidates in the pharmaceutical, and cosmetic industries [2]. Indeed, the production of bacterial polysaccharides has several advantages: It can be easily controlled and is independent of seasonal variation or climatic events. The polymers produced are reproducible and can be very highly purified. It all makes them appropriate for niche applications in the biomedical sector. In addition, polysaccharides can be chemically modified (sulfation or phosphorylation) to impart or improve particular functional properties, further increasing their potential for use.

With the discovery of new polysaccharides of biotechnological interest, extremophilic microorganisms have been widely recognized as an industrial source of great importance for application in various processes and a model for investigating the role of their biomolecules in bacterial adaptation to extreme conditions. To answer the question of how metabolites are stabilized under extreme conditions, it is necessary to expand fundamental knowledge about the structural diversity of bioglycans produced by extremophilic bacteria, including deep-sea bacteria.

The study presented herein is a part of a project on the systematic structural investigation of polysaccharides produced by deep-sea Gram-negative bacteria. Previously, we elucidated the structures of the O-polysaccharides and CPS from *Rheinheimera pacifica*, *Idiomarina abyssalis*, *Pseudomonas stutzeri*, and *Psychrobacter submarinus* [5,6,7,8]. In this paper, we report on the isolation, purification, and structure of the cell-wall-associated polysaccharides produced by the bacterium *Devosia submarina* KMM 9415^T^ [9], isolated from a surface sediment sample at a depth of 515 m. To date, there is no information on the polysaccharides produced by bacteria of the genus *Devosia*.

## 2. Results

### 2.1. Isolation, Purification, and General Characterization of CPS

**Figure 1:**
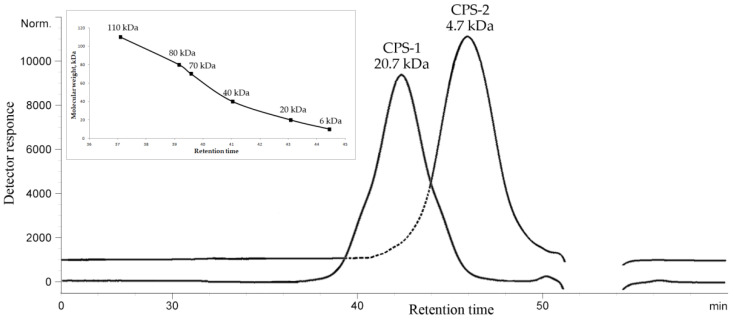
HPSEC elution profiles of the CPS-1 and CPS-2 fractions from *D. submarina* KMM 9415^T^, and dextran standards calibration curve (insert).

The dried bacterial cells were subjected to the extraction with a saline solution, followed by ultracentrifugation and enzymatic treatment, and the resulting CPS-containing material was further purified by anion-exchange chromatography and hydrophobic interaction chromatography. The SDS–PAGE of the purified CPS preparation revealed no ladder-like bands typical for LPS contamination. The CPS preparation was fractionated by gel permeation chromatography, and as a result, two polysaccharide fractions (CPS-1 and CPS-2) were obtained in a ratio of about 1.8:1. The size exclusion chromatography (SEC) analysis showed that the CPS-1 and CPS-2 fractions formed single peaks and had a molecular weight of about 20.7 kDa and 4.7 kDa, respectively (Figure 1). 

**Figure 2:**
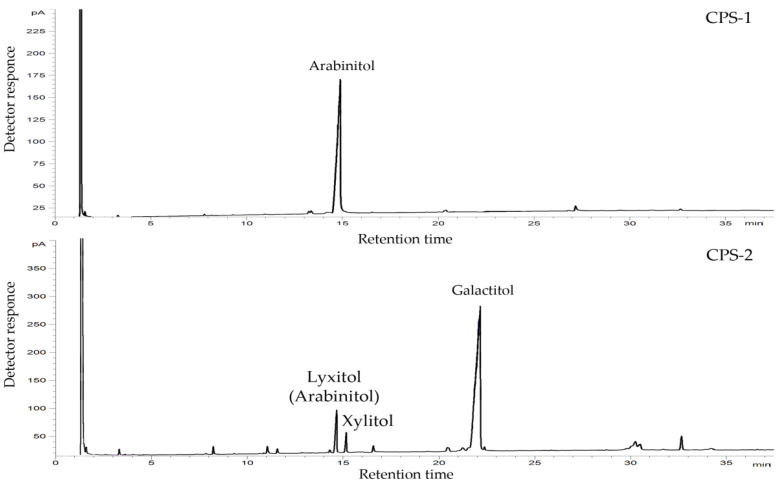
GC profiles of the acetylated alditols derived from CPS-1 (**top**) and CPS-2 (**bottom**).

Using monosaccharide analysis by GC of acetylated alditols obtained after the complete acid hydrolysis of polysaccharides, arabinose (Ara) (CPS-1), galactose (Gal), and xylulose (Xlu) (CPS-2) were identified. The latter monosaccharide produced two alditols, xylitol, and lyxitol (arabinitol), upon reduction (Figure 2). The D absolute configurations of Ara and Gal were determined by GC of the acetylated (*S*)-2-octyl glycosides. The D absolute configuration of Xlu was determined by calculation of the specific optical rotation of the disaccharide fragment obtained by mild acid hydrolysis of CPS-2 using the Klyne rule (see below).

### 2.2. Structural Study of the CPS-1 Sample

**Figure 3:**
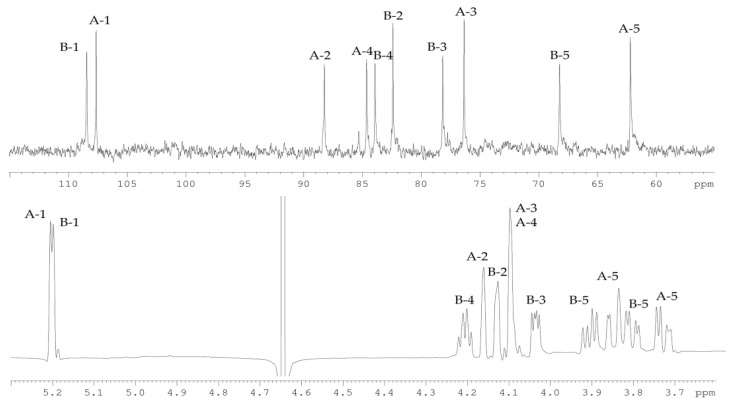
^13^C (**top**) and ^1^H (**bottom**) NMR spectra of CPS-1. Numerals refer to carbons and protons in sugar residues denoted by capital letters as described in Table 1.

The ^13^C NMR spectrum (Figure 3) of the CPS-1 sample displayed signals of two anomeric carbons at *δ_C_* 107.6, and 108.4, two hydroxymethyl groups at *δ_C_* 62.2, and 68.2, as well as six other sugar ring carbons in the region of *δ_C_* 76.3–88.2. Accordingly, the ^1^H NMR spectrum (Figure 3) of CPS-1 showed signals for two anomeric protons at *δ_H_* 5.20, and 5.19, and other sugar protons at *δ_H_* 3.73–4.21. Two signals in the anomeric region, together with a total number of signals in the ^13^C NMR spectrum, demonstrated the disaccharide repeating unit of CPS-1, composed of d-Ara residues.

**Figure 4:**
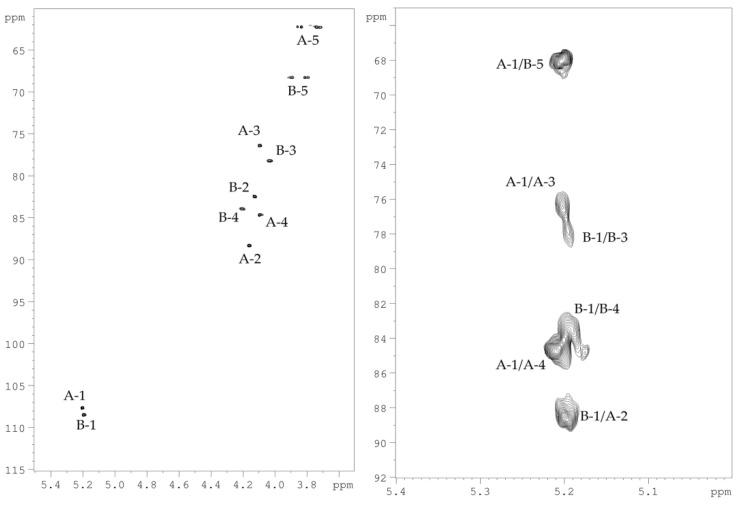
^1^H,^13^C-HSQC (**left**) and a part of ^1^H,^13^C-HMBC (**right**) NMR spectra of CPS-1. Numerals refer to protons and carbons in sugar residues denoted by capital letters as described in Table 1.

The spin-systems of sugar residues were assigned by ^1^H,^1^H-COSY, 1D-TOCSY experiments. From the assigned ^1^H signals and the one-bond C-H connectivities, the carbon signals were assigned in the ^1^H,^13^C-HSQC spectrum (Figure 4). The monosaccharide residues in CPS-1 were arbitrarily labeled **A** and **B**, according to decreasing chemical shift values of their anomeric protons (Table 1). The furanose form and α-configuration of both sugar residues were identified by comparing chemical shifts with the published NMR data for the individual monosaccharides [10]. The substitution positions of the respective monosaccharides were identified based on the relatively low-field chemical shift values of the substituted carbons, compared with values for the unsubstituted monosaccharide [10]. Residue **A** with H1/C1 signals at *δ* 5.20/107.6 was recognized as 2-substituted α-d-Ara*f* based on the low-field chemical shift of the C-2 signal (*δ_C_* 88.2). Residue **B** with H1/C1 signals at δ 5.23/108.3 was identified as 5-substituted α-d-Ara*f* due to the characteristic low-field chemical shifts of the C-5 signal (*δ_C_* 68.2).

The sequence of the monosaccharide residues within the repeating unit of CPS-1 was confirmed using the ^1^H,^1^H-ROESY, and ^1^H,^13^C-HMBC experiments. The following inter-residue correlations between anomeric protons and protons at the linkage carbons were observed in the ^1^H,^1^H-ROESY spectrum of the CPS-1 sample: **A** H-1/**B** H-5 at *δ* 5.20/3.91, 3.80; **B** H-1/**A** H-2 at *δ* 5.19/4.16. The ^1^H,^13^C-HMBC experiment (Figure 4) demonstrated the respective correlations between the following anomeric protons and the carbon at the linkage position: **A** H-1/**B** C-5 at *δ* 5.20/68.2; **B** H-1/**A** C-2 at *δ* 5.19/88.2. 

Thus, the combined results showed the disaccharide repeating unit of the CPS-1 of *D. submarina* KMM 9415^T^ with the following structure:

→2)-α-d-Ara*f*-(1→5)-α-d-Ara*f*-(1→

### 2.3. Structural Study of the CPS-2 Sample

**Figure 5:**
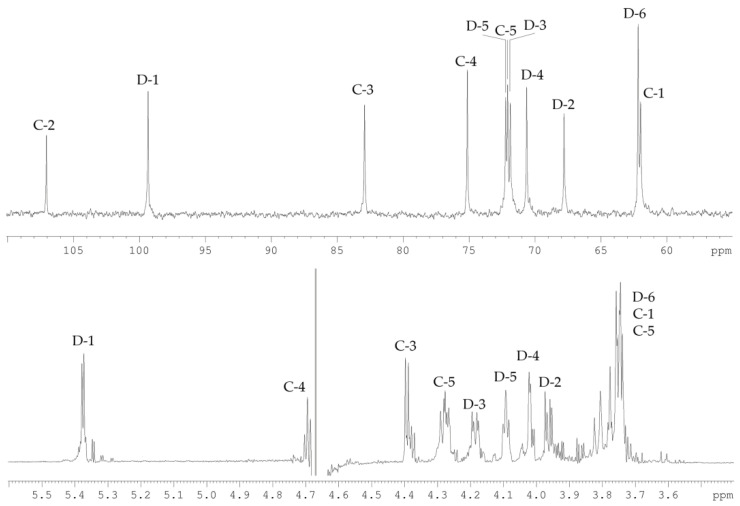
^13^C (**top**) and ^1^H (**bottom**) NMR spectra of the CPS-2 sample. Numerals refer to carbons and protons in sugar residues denoted by capital letters as described in Table 2.

The ^13^C NMR spectrum (Figure 5) of the CPS-2 sample contained signals of two anomeric carbons at *δ_C_* 99.3, and 107.0, three hydroxymethyl groups at *δ_C_* 62.0, 62.2, and 72.1, as well as six other sugar ring carbons in the region of *δ_C_* 67.8–82.9. In turn, the ^1^H NMR spectrum (Figure 5) of CPS-2 showed signals for only one anomeric proton at *δ_H_* 5.38 and other sugar protons in the region of *δ_H_* 3.75–4.69. The signals belonged to two different monosaccharide residues, one of which was aldose (d-Gal*p*, H-1/C-1 at *δ* 5.38/99.3). In the other monosaccharide, the anomeric proton was absent, and the signal at *δ_C_* 107.0 belonged to the unprotonated anomeric carbon of ketose (C-2 of Xlu*f*). 

**Figure 6:**
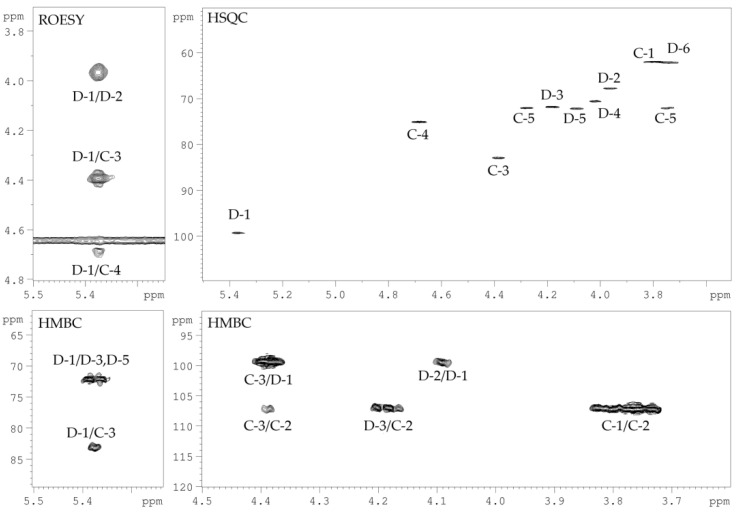
Sections of ^1^H,^1^H-ROESY, ^1^H,^13^C-HSQC (**top**) and, ^1^H,^13^C-HMBC (**bottom**) spectra of the CPS-2 sample. Numerals refer to carbons and protons in sugar residues denoted by capital letters as described in Table 2.

The signals in the NMR spectra were assigned using ^1^H,^1^H-COSY, 1D TOCSY, ^1^H,^13^C-HSQC, and ^1^H,^13^C-HMBC experiments (Figure 6, Table 2). 

The sugar residues in the CPS-2 sample were labeled **C** and **D**, according to decreasing chemical shift values of their anomeric carbons (Table 2). Analysis of the correlations from H-3 to H-5 in the ^1^H,^1^H-COSY spectrum in combination with the total number of NMR signals and the presence of two hydroxymethyl groups (H-1/C-1 at *δ* 3.71, 3.76/62.0 and H-5/C-5 at *δ* 3.75, 4.28/72.1) showed that the ketose was a pent-2-ulose. A comparison of its chemical shifts with literature data for methyl-α- and methyl-β-d-*threo*-pent-2-ulofuranosides, together with data of sugar analysis, showed that ketose had the β-*threo* configuration (xylulose) [11]. 

Another monosaccharide present was identified as d-Gal by the correlations from H-1 to H-4, H-5 to H-6 in the 1D TOCSY spectrum, and the characteristic spin–spin coupling constants ^3^*J*_3,4_ < 4 Hz. The d-Gal was in the pyranose form and was linked by an α-glycosidic bond, which followed from the small constant ^3^*J*^1,2^ (3.2 Hz), the chemical shift *δ_C_* 72.2 for C-5, as well as from the strong correlations between d-Gal*p* H-1 and H-2 atoms revealed by the ^1^H,^1^H-ROESY spectrum, with the absence of correlations between H-1 and H-3, H-5 atoms.

The ^1^H,^1^H-ROESY spectrum (Figure 6) contains the correlation peaks between the H-1 atom of d-Gal*p* and H-3, H-4 atoms of Xlu*f*. A comparison of ^13^C chemical shifts showed that Xlu*f* C-3 atom resonated in lower field at *δ_C_* 83.0, while C-4 atom in a higher field at *δ_C_* 75.2, as compared with that of Me-β-Xlu*f* [11]. Thus, Xlu*f* was substituted at position O-3. An α-glycosylation effect of 1.6 ppm was observed for the C-3 atom of d-Gal*p.* Such a small α-effect is typical of glycosylation with a ketose and indicates the substitution of d-Gal*p* by Xlu*f* at position O-3. These findings were confirmed in the ^1^H,^13^C-HMBC experiment (Figure 6) by the presence of correlation between C-2 of Xlu*f* and H-3 of d-Gal*p* at *δ* 107.0/4.19. 

**Figure 7:**
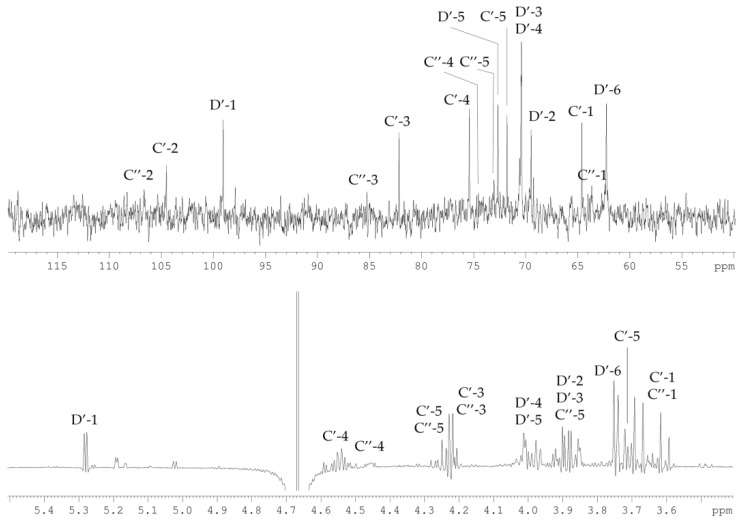
^13^C (**top**) and ^1^H (**bottom**) NMR spectra of the OS. Numerals refer to carbons and protons in sugar residues denoted by capital letters as described in Table 3.

To confirm the structure, CPS-2 was subjected to mild acid hydrolysis, which resulted in cleavage of the glycosidic bond between Xlu*f* and d-Gal*p*. As expected, the disaccharide fraction (OS) with Xlu*f* on reducing end was obtained, whose structure was established by 1D (Figure 7) and 2D NMR spectroscopy as described above (Table 3). 

The absolute configuration of Xlu*f* was determined by calculation of the specific optical rotation of OS using the Klyne rule [12]. The calculation showed that Xlu*f* had the d-configuration (the experimental value for OS [α]_D_ +36 (water); the calculated values [α]_D_ +30 and +60 for the OS with d-Xlu*f* and L-Xlu*f*, respectively).

Based on the data obtained, it was concluded that CPS-2 had the following structure:

→3)-α-d-Gal*p*-(1→3)-β-d-Xlu*f*-(1→

## 3. Discussion

Most bacterial glycans are high molecular weight heteropolysaccharides containing three or four different monosaccharides arranged in groups of ten or less to form repeating units. Some polysaccharides are neutral macromolecules, but most are polyanionic due to the presence of uronic acids and charged non-carbohydrate substituents. It is believed that polysaccharides produced by microorganisms from extreme habitats show biomedical promise due to their antiviral and immunomodulatory effects, bone regeneration, and cicatrizing capacity [2,13].

**Figure 8:**
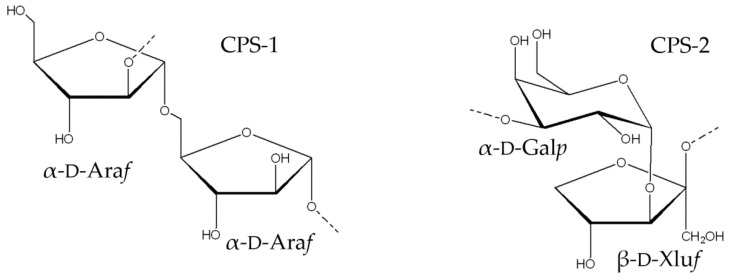
Structures of the CPS-1 and CPS-2 of *D. submarina* KMM 9415^T^.

In this study, we determined the structures of the two cell-wall-associated polysaccharides isolated from the marine deep-sea bacterium *D. submarina* KMM 9415^T^ (Figure 8). The first polysaccharide of the strain KMM 9415^T^ consists of disaccharide repeating units, composed of α-d-arabinofuranoside residues. The second polysaccharide also consists of disaccharide repeating units, composed of α-d-galactopyranoside and β-d-*threo*-pent-2-ulofuranoside (xylulose) residues. To our knowledge, these structures have not previously been found [14].

Lipoarabinomannans are the glycolipid antigens found commonly in all mycobacterial cell walls and are interesting vaccine candidates to evoke immune responses against *Mycobacterium tuberculosis* [15]. L-arabinofuranans are often found in the fruit bodies (or rinds) of certain plants and exhibit immunomodulatory properties. Polysaccharides significantly stimulate phagocytic activity and improve the secreted level of nitric oxide, interleukin-6, interleukin-1β, and tumor necrosis factor-α of macrophages [16,17]. Xylulose is a rather rare component of bacterial polysaccharides. To date, it has been found in O-polysaccharides of *Escherichia coli* O95 [18], *Yersinia enterocolitica* 0:10 [19], as well as in the CPS from *Campylobacter jejuni* RM1221 [11] and *Eubacterium saburreum* T15 [20].

Both polysaccharides are neutral, which is not typical of marine bacteria [2,13]. At the same time, the results indicate that some neutral polysaccharides from extremophilic bacteria possess immunomodulatory and antiviral effects. [21,22,23]. It is not uncommon for Gram-negative bacteria to produce more than one polysaccharide. The presence of several different polysaccharides has previously been found in other microorganisms [24,25,26,27,28,29,30,31,32,33,34,35,36,37,38]. In the case of *D. submarina* KMM 9415^T^, the ability to produce structurally different polysaccharides may be useful for adaptation to the parameters of the deep-sea environment. 

## 4. Materials and Methods

### 4.1. Isolation and Purification of CPSs

*D. submarina* KMM 9415^T^ was obtained from the Collection of Marine Microorganisms (KMM) of the G.B. Elyakov Pacific Institute of Bioorganic Chemistry, Far Eastern Branch of Russian Academy of Sciences (Vladivostok, Russia). The bacteria were cultivated for 48 h at ambient temperature on a medium composed of (L^−1^): 5.0 g Bacto peptone (Difco), 2 g Bacto yeast extract (Difco), 0.2 g KH_2_PO_4_ 1.0, g glucose, and 0.05 g MgSO_4_ in 50% (*v/v*) distilled water and 50% (*v/v*) natural seawater. Dry bacterial cells (2.9 g) were suspended in 50 mL of extraction buffer (0.22 M NaCl, 0.026 M MgCl_2_, 0.01 M KCl) and kept stirring for 16 h at 4 °C. The cell pellet was collected by centrifugation (5000 rpm, 25 min, 4 °C) and the extraction procedure was repeated two more times (4 °C, 1 h). Supernatants were combined, deproteinized by the Sevag method, dialyzed (MWCO 3500 Da), and lyophilized to yield crude CPS (49.3 mg). The freeze-dried material was resuspended in 5 mL of digestion buffer (pH 7.5) containing TRIS/EDTA (0.01 M/0.001 M) and 0.01 M MgCl_2_. After the addition of 1 mg of proteinase K (Sigma-Aldrich, St. Louis, MO, USA), the solution was incubated for 2 h at 60 °C. After dialysis against distilled water (MWCO 3500 Da), it was lyophilized to give enzymatic treated CPS (20.2 mg). The material was resuspended in 2 mL of water and centrifuged at 108,000× *g* for 4 h on Avanti JXN-30 (Beckman Coulter, Pasadena, CA, USA). The freeze-dried supernatant was purified by hydrophobic interaction chromatography using a Butyl Sepharose 4FF column (10 cm × 1.5 cm, Sigma-Aldrich, USA) with a method described [39]. The CPS was eluted with buffer in a non-bound fraction. After dialysis and lyophilization, polysaccharide material was subjected to anion-exchange chromatography on a column (10 cm × 1.5 cm) of Toyopearl DEAE-650M (Tosoh Bioscience, Tokyo, Japan) in a step-wise gradient of NaCl. The resulting main neutral fraction (18.1 mg) was dialyzed and freeze-dried. Finally, the CPS was loaded on the *Toyopearl HW-40 column* (120 cm × 1.5 cm, Tosoh Bioscience, Tokyo, Japan) eluted with aq 0.1% AcOH, yielding CPS-1 (11.2 mg) and CPS-2 (6.1 mg). Elution was monitored with a differential refractometer (Knauer, Germany).

### 4.2. Determination of the Molecular Weight of CPSs

Molecular weights of CPS-1 and CPS-2 samples were analyzed using HPLC (Agilent 1100 Series, Hamburg, Germany), equipped with successively connected columns of Shodex Asahipak GS-520 HQ and GS-620 HQ (7.5 mm × 300 mm) at 50 °C with elution by 0.15 M NaCl (0.4 mL/min). Columns were calibrated using standard dextrans of 6, 20, 40, 70, 80, and 110 kDa (Sigma, St. Louis, MO, USA).

### 4.3. Compositional Analysis of the CPS Samples

Monosaccharides were analyzed as the alditol acetates obtained by hydrolysis of the CPS-1 (2 M CF_3_COOH, 120 °C, 2 h) and CPS-2 (0.5 M CF_3_COOH, 100 °C, 3 h) by GC on an Agilent 6850 chromatograph (Santa Clara, CA, USA) equipped with an HP-5 MS capillary column using a temperature program from 150 °C (3 min) to 230 °C (10 min) at 3 °C min^−1^. The absolute configurations of monosaccharides were determined by GC of the acetylated (*S*)-2-octyl glycosides as described [40]. The absolute configuration of d-Xlu was determined by calculation of the specific optical rotation of OS using the Klyne rule [12]. The specific optical rotation of OS was measured on a PerkinElmer instrument, model 343 (Waltham, MA, USA). Fatty acid analysis was performed by GC of methyl derivatives after methanolysis of CPS with 2 M acetylchloride in methanol (120 °C, 4 h). Proteins were analyzed by the conventional method [41].

### 4.4. Mild Acid Hydrolysis of CPS-2

The CPS-2 sample (4 mg) was hydrolyzed with 2% AcOH (0.8 mL) for 2 h at 100 °C. The product was fractioned by gel permeation chromatography on TSK HW-40 (120 cm × 1.5 cm, Tosoh Bioscience, Tokyo, Japan) in aq 0.1% AcOH to give the OS (2.8 mg). Elution was monitored with a differential refractometer (Knauer, Berlin, Germany).

### 4.5. NMR Spectroscopy

^1^H and ^13^C NMR spectra of CPS-1, CPS-2, and OS were recorded on a Bruker Avance-III (700.13 MHz for ^1^H and 176.04 MHz for ^13^C) spectrometer (Germany) at 35 °C using acetone (δ_C_ 31.45, δ_H_ 2.225) as internal standard. The 2D NMR spectra were obtained using standard Bruker software, and Bruker TOPSPIN 2.1 program (Bruker Corporation, Billerica, MA, USA) was used to acquire and process the NMR data. The 1D TOCSY and ^1^H,^1^H-ROESY spectra were recorded with a 180 ms duration of MLEV-17 spin-lock and a 200 ms mixing time, respectively. ^1^H,^13^C-HMBC was optimized for an 8 Hz long-range constant.

## 5. Conclusions

Bioglycans of extremophilic microorganisms often have unique properties, but their structural characteristic and functional roles have not been adequately studied [2]. The study of the structures of extracellular and membrane polysaccharides can provide a deeper understanding of both the role of these bioglycans in the adaptation of bacteria to extreme conditions and predict the possibility of their use for biotechnological purposes.

## Figures and Tables

**Table 1:** The ^1^H and ^13^C NMR data for CPS-1, (δ, ppm).

Sugar Residue	H-1	H-2	H-3	H-4	H-5
	C-1	C-2	C-3	C-4	C-5
→2)-α-d-Ara*f*-(1→	5.20	4.16	4.10	4.10	3.85, 3.73
**A**	107.6	88.2	76.3	84.6	62.2
→5)-α-d-Ara*f*-(1→	5.19	4.13	4.04	4.21	3.91, 3.80
**B**	108.4	82.4	78.2	83.9	68.2

**Table 2:** ^1^H and ^13^C NMR data for the CPS-1 sample, (*δ*, ppm).

Sugar Residue	H-1	H-2	H-3	H-4	H-5	H-6
	C-1	C-2	C-3	C-4	C-5	C-6
→3)-β-d-Xlu*f*-(2→	3.71, 3.76		4.39	4.69	3.75, 4.28	
**C**	62.0	107.0	83.0	75.2	72.1	
→3)-α-d-Gal*p*-(1→	5.38	3.96	4.19	4.02	4.09	3.75, 3.75
**D**	99.3	67.8	71.9	70.6	72.2	62.2

**Table 3:** ^1^H and ^13^C NMR data for the OS sample, (*δ*, ppm).

Sugar Residue	H-1	H-2	H-3	H-4	H-5	H-6
	C-1	C-2	C-3	C-4	C-5	C-6
α-d-Gal*p*-(1→	5.28	3.89	3.92	4.01	3.98	3.75, 3.75
**D’**	99.1	69.5	70.4	70.4	72.6	62.2
→3)-β-d-Xlu*f*	3.60, 3.68		4.22	4.55	3.71, 4.23	
**C’**	64.6	104.5	82.1	75.4	71.8	
→3)-α-d-Xlu*f*	3.71, 3.71		4.21	4.46	3.91, 4.26	
**C’’**	63.7	106.6	85.3	74.6	73.1	

## References

[1] Zhang Z., Wu Y., Zhang X.-H. (2018). Cultivation of microbes from the deep-sea environments. Deep Sea Res. Part II Top. Stud. Oceanogr..

[2] Casillo A., Lanzetta R., Parrilli M., Corsaro M.M. (2018). Exopolysaccharides from marine and marine extremophilic bacteria: Structures, properties, ecological roles and applications. Mar. Drugs.

[3] Wang Y.-N., Meng L.-H., Wang B.-G. (2020). Progress in Research on Bioactive Secondary Metabolites from Deep-Sea Derived Microorganisms. Mar. Drugs.

[4] Herget S., Toukach P.V., Ranzinger R., Hull W.E., Knirel Y.A., Von der Lieth C.-W. (2008). Statistical analysis of the Bacterial Carbohydrate Structure Data Base (BCSDB): Characteristics and diversity of bacterial carbohydrates in comparison with mammalian glycans. BMC Struct. Biol..

[5] Komandrova N.A., Kokoulin M.S., Kalinovsky A.I., Tomshich S.V., Romanenko L.A., Vaskovsky V.E. (2014). The O-specific polysaccharide of the marine bacterium *Rheinheimera pacifica* KMM 1406^T^ containing d- and L-2-acetamido-2-deoxy-galacturonic acids. Carbohydr. Res..

[6] Kokoulin M.S., Komandrova N.A., Kalinovsky A.I., Tomshich S.V., Romanenko L.A., Vaskovsky V.E. (2015). Structure of the O-specific polysaccharide from the deep-sea marine bacterium *Idiomarina abyssalis* KMM 227^T^ containing a 2-*O*-sulfate-3-*N*-(4-hydroxybutanoyl)-3,6-dideoxy-d-glucose. Carbohydr. Res..

[7] Kokoulin M.S., Sokolova E.V., Elkin Y.N., Romanenko L.A., Mikhailov V.V., Komandrova N.A. (2017). Partial structure and immunological properties of lipopolysaccharide from marine-derived *Pseudomonas stutzeri* KMM 226. Antonie Van Leeuwenhoek.

[8] Kokoulin M.S., Kuzmich A.S., Romanenko L.A., Chikalovets I.V. (2021). Structure and in vitro antiproliferative activity of the acidic capsular polysaccharide from the deep-sea bacterium *Psychrobacter submarinus* KMM 225^T^. Carbohydr. Polym..

[9] Romanenko L.A., Tanaka N., Svetashev V.I. (2013). *Devosia submarina* sp. nov., isolated from deep-sea surface sediments. Int. J. Syst. Evol. Micr..

[10] Bock K., Pedersen C. (1983). Carbon-13 nuclear magnetic resonance spectroscopy of monosaccharides. Adv. Carbohydr. Chem. Biochem..

[11] Gilbert M., Mandrell R.E., Parker C.T., Li J., Vinogradov E. (2007). Structural analysis of the capsular polysaccharide from Campylobacter jejuni RM1221. ChemBioChem.

[12] Klyne J. (1950). The Configuration of the Anomeric Carbon Atoms in some Cardiac Glycosides. Biochem. J..

[13] Poli A., Anzelmo G., Nicolaus B. (2010). Bacterial exopolysaccharides from extreme marine habitats: Production, characterization and biological activities. Mar. Drugs.

[14] Toukach P.V., Egorova K.S. (2016). Carbohydrate structure database merged from bacterial, archaeal, plant and fungal parts. Nucleic Acids Res..

[15] Leelayuwapan H., Ruchirawat S., Boonyarattanakalin S. (2019). Rapid synthesis and immunogenicity of mycobacterial (1→5)-α-d-arabinofuranan. Carbohydr. Polym..

[16] Zhang S., Li Z., Wang X., An L., Bao J., Zhang J., Cui J., Li Y., Jin D.-Q., Tuerhong M. (2020). Isolation, structural elucidation, and immunoregulation properties of an arabinofuranan from the rinds of *Garcinia mangostana*. Carbohydr. Polym..

[17] Wang H., Wang X., Li Y., Zhang S., Li Z., Li Y., Cui J., Lan X., Zhang E., Yuan L. (2021). Structural properties and in vitro and in vivo immunomodulatory activity of an arabinofuranan from the fruits of *Akebia quinata*. Carbohydr. Polym..

[18] Perepelov A.V., Senchenkova S.N., Kalinchuk N.A., Shashkov A.S., Knirel Y.A. (2018). Structure of O-polysaccharide of *Escherichia coli* O95: A disaccharide repeating unit containing d-fucose and d-threo-pent-2-ulose (xylulose). Russ. Chem. Bull..

[19] Gorshkova R.P., Isakov V.V., Kalmykova E.N., Ovodov Y.S. (1995). Structural studies of O-specific polysaccharide chains of the lipopolysaccharide from *Yersinia enterocolitica* serovar O:10. Carbohydr. Res..

[20] Sato N., Nakazawa F., Ito T., Hoshino T., Hoshino E. (2003). The structure of the antigenic polysaccharide produced by *Eubacterium saburreum* T15. Carbohydr. Res..

[21] Arena A., Gugliandolo C., Stassi G., Pavone B., Iannello D., Bisignano G., Maugeri T.L. (2009). An exopolysaccharide produced by *Geobacillus thermodenitrificans* strain B3-72: Antiviral activity on immunocompetent cells. Immunol. Lett..

[22] Arena A., Maugeri T.L., Pavone B., Iannello D., Gugliandolo C., Bisignano G. (2006). Antiviral and immunoregulatory effect of a novel exopolysaccharide from a marine thermotolerant Bacillus licheniformis. Int. Immunopharmacol..

[23] Kambourova M., Mandeva R., Dimova D., Poli A., Nicolaus B., Tommonaro G. (2009). Production and characterization of a microbial glucan, synthesized by Geobacillus tepidamans V264 isolated from Bulgarian hot spring. Carbohydr. Polym..

[24] Cox A.D., Taylor C.J., Anderson A.J., Perry M.B., Wilkinson S.G. (1995). Structures of the two Polymers Present in the Lipopolysaccharide of *Burkholderia (Pseudomonas) Cepacia* Serogroup O4. Eur. J. Biochem..

[25] Cerantola S., Montrozier H. (1997). Structural elucidation of two polysaccharides present in the lipopolysaccharide of a clinical isolate of *Burkholderia cepacia*. Eur. J. Biochem..

[26] Lipinski T., Zatonsky G.V., Kocharova N.A., Jaquinod M., Forest E., Shashkov A.M., Gamian A., Knirel Y.A. (2002). Structures of two O-chain polysaccharides of *Citrobacter gillenii* O9a,9b lipopolysaccharide: A new homopolymer of 4-amino-4,6-dideoxy-d-mannose (perosamine) *Eur*. J. Biochem..

[27] De Castro C., Molinaro A., Wallace A., Grant W.D., Forest E., Parrilli M. (2003). The O-specific chain structure of the major component from the lipopolysaccharide fraction of *Halomonas magadii* strain 21 MI (NCIMB 13595). Eur. J. Org. Chem..

[28] De Castro C., Carannante A., Lanzetta R., Nunziata R., Piscopo V., Parrilli M. (2004). Elucidation of two O-chain structures from the lipopolysaccharide fraction of *Agrobacterium tumefaciens* F/1. Carbohydr. Res..

[29] Ovchinnikova O.G., Kocharova N.A., Katzenellenbogen E., Zatonsky G.V., Shashkov A.M., Knirel Y.A., Lipinski T., Gamian A. (2004). Structures of two O-polysaccharides of the lipopolysaccharide of *Citrobacter youngae* PCM 1538 (serogroup O9). Carbohydr. Res..

[30] Zdorovenko E.L., Varbanets L.D., Zatonsky G.V., Ostapchuk A.N. (2006). Structures of two putative O-specific polysaccharides from the *Rahnella aquatilis* 3-95 lipopolysaccharide. Carbohydr. Res..

[31] Fernandez de Cordoba F.J., Rodriguez-Carvajal M.A., Tejero-Mateo P., Corzo J., Gil-Serrano A.M. (2008). Structure of the O-Antigen of the Main Lipopolysaccharide Isolated from *Sinorhizobium fredii* SMH12. Biomacromolecules.

[32] Zdorovenko E.L., Varbanets L.D., Zatonsky G.V., Zdorovenko G.M., Shashkov A.M., Knirel Y.A. (2009). Isolation and structure elucidation of two different polysaccharides from the lipopolysaccharide of *Rahnella aquatilis* 33071^T^. Carbohydr. Res..

[33] Ierano T., Silipo A., Cescutti P., Leone M.R., Rizzo R., Lanzetta R., Parrilli M., Molinaro A. (2009). Structural Study and Conformational Behavior of the Two Different Lipopolysaccharide O-Antigens Produced by the Cystic Fibrosis Pathogen *Burkholderia multivorans*. Chem. Eur. J..

[34] Komandrova N.A., Isakov V.V., Tomshich S.V., Romanenko L.A., Perepelov A.V., Shashkov A.S. (2010). Structure of an acidic O-specific polysaccharide of the marine bacterium *Pseudoalteromonas agarivorans* KMM 232 (R-form). Biochem. Mosc..

[35] Silipo A., Leone M.R., Lanzetta R., Parrilli M., Lackner G., Busch B., Hertweck C., Molinaro A. (2012). Structural characterization of two lipopolysaccharide O-antigens produced by the endofungal bacterium *Burkholderia* sp. HKI-402 (B4). Carbohydr. Res..

[36] Sigida E.N., Fedonenko Y.P., Shashkov A.S., Zdorovenko E.L., Konnova S.A., Ignatov V.V., Knirel Y.A. (2015). Structure of the polysaccharides from the lipopolysaccharide of *Azospirillum brasilense* Jm125A2. Carbohydr. Res..

[37] Kokoulin M.S., Tomshich S.V., Kalinovsky A.I., Romanenko L.A., Komandrova N.A. (2016). Structure of polysaccharide moiety of *Pseudomonas xanthomarina* KMM 1447^T^ lipopolysaccharide. Carbohydr. Res..

[38] Kokoulin M.S., Lizanov I.N., Romanenko L.A., Chikalovets I.V. (2020). Structure of phosphorylated and sulfated polysaccharides from lipopolysaccharide of marine bacterium *Marinicella litoralis* KMM 3900^T^. Carbohydr. Res..

[39] Ren K., Li Y., Shi F., Wang X. (2012). Separation of lipopolysaccharides containing different fatty acid chains using hydrophobic interaction chromatography. Anal. Methods.

[40] Leontein K., Lindberg B., Lönngren J. (1978). Assignment of absolute configuration of sugars by g.l.c. of their acetylated glycosides formed from chiral alcohols. Carb. Res..

[41] Bradford M.M. (1976). A rapid and sensitive method for the quantitation of microgram quantities of protein utilizing the principle of protein-dye binding. Anal. Biochem..

